# Evaluation of RECIST in chemotherapy-treated lung cancer: the Pharmacogenoscan Study

**DOI:** 10.1186/1471-2407-14-989

**Published:** 2014-12-20

**Authors:** Anne-Claire Toffart, Denis Moro-Sibilot, Sébastien Couraud, Patrick Merle, Maurice Perol, Nicolas Girard, Pierre-Jean Souquet, Bénédicte Mastroianni, Gilbert R Ferretti, Philippe Romand, Patrick Chatellain, Aurélien Vesin, Elisabeth Brambilla, Christian Brambilla, Jean-François Timsit

**Affiliations:** Université Grenoble 1 INSERM U 823-Institut A Bonniot-Université J Fourier, Rond-point de la Chantourne, 38706 La Tronche Cedex, France; Thoracic Oncology Unit, Centre Hospitalier Universitaire A Michallon, 2-BP 217, 38043 Grenoble cedex, France; Pulmonology Department, Centre Hospitalier Lyon Sud, Hospices Civils de Lyon, 165 chemin du grand Revoyet, 69495 Pierre Bénite cedex, France; Lyon Sud Medical Faculty, Lyon 1 University, 165 chemin du Petit Revoyet, BP 12, 69921 Oullins cedex, France; Respiratory Medicine – Thoracic Oncology Unit, Centre Hospitalier Universitaire G. Montpied, 58 Rue Montalembert, 63003 Clermont Ferrand Cedex 1, France; EA 7283- Université d’Auvergne, INSERM CIC 501, Clermont-Ferrand, France; Thoracic Oncology Unit, Croix-Rousse Hospital, Hospices Civils de Lyon, 103 Grande Rue de la Croix-Rousse, 69317 Lyon cedex 04, France; Respiratory Medicine Service, Hôpital Louis Pradel, Hospices Civils de Lyon, 28 avenue doyen Lépine, 69677 Lyon-Bron cedex, France; UMR 754, Université Claude Bernard Lyon 1, 50 avenue Tony Garnier, 69007 Lyon, France; Radiology and Medical Imaging Unit, Centre Hospitalier Universitaire A Michallon, BP 217, 38043 Grenoble cedex 9, France; Respiratory Medicine Service, Hôpitaux du Leman, 3 avenue de la Dame, BP 526, 74203 Thonon Les Bains, France; Respiratory Medicine Service, Centre Hospitalier Alpes Leman, 558 route de Findrol, BP 20 500, 74130 Contamine sur Arve, France; Department of Pathology, Centre Hospitalier Universitaire A Michallon, BP 217, 38043 Grenoble cedex 9, France; ThoracicOncology Unit, Centre Hospitalier Universitaire A Michallon, BP 217, 38043 Grenoble cedex 9, France; Medical Intensive Care Unit, Centre Hospitalier Universitaire A Michallon, BP 217, 38043 Grenoble cedex 9, France

**Keywords:** Lung neoplasm, Drug response, RECIST, Survival, Cox model, Akaike information criteria

## Abstract

**Background:**

Response Evaluation Criteria in Solid Tumors (RECIST) are widely used to assess the effect of chemotherapy in patients with cancer. We hypothesised that the change in unidimensional tumour size handled as a continuous variable was more reliable than RECIST in predicting overall survival (OS).

**Methods:**

The prospective Pharmacogenoscan study enrolled consecutive patients with non-small-cell lung cancer (NSCLC) at any stage seen between 2005 and 2010 at six hospitals in France, given chemotherapy. After exclusion of patients without RECIST or continuous-scale tumour size data and of those with early death, 464 patients were left for the survival analyses. Cox models were built to assess relationships between RECIST 1.1 categories or change in continuous-scale tumour size and OS. The best model was defined as the model minimising the Akaike Information Criterion (AIC).

**Results:**

OS was 14.2 months (IQR, 7.3-28.9 months). According to RECIST 1.1, 146 (31%) patients had a partial or complete response, 245 (53%) stable disease, and73 (16%) disease progression. RECIST 1.1 predicted better OS than continuous-scale tumour in early (<6 months) predicted survival analyses (*p* = 0.03) but the accuracy of the two response evaluation methods was similar in late (≥6 months) predicted survival analyses (*p* = 0.15).

**Conclusion:**

In this large observational study, change in continuous-scale tumour size did not perform better than RECIST 1.1 in predicting survival of patients given chemotherapy to treat NSCLC.

**Trial registration:**

NCT00222404

## Background

Response Evaluation Criteria in Solid Tumors (RECIST) was developed in 2000 [1] to assess changes in solid tumour size in patients given cancer chemotherapy. RECIST criteria are based on the sum of the maximum diameters of target lesions seen on imaging studies. This value is categorised as follows: complete/partial response (CR/PR), complete disappearance of all targets/greater than 30% decrease; stable disease (SD), change between −30% and +20%; and progressive disease (PD), greater than 20% increase. The initial RECIST guidelines (RECIST 1.0) were revised in 2009 (RECIST 1.1) [2] to improve the definitions of the target lesions. RECIST categories have gradually superseded the World Health Organisation (WHO) criteria for chemotherapy effects, which use bidimensional tumour measurements to define CR, PR, SD, and PD [3]. RECIST categories were found to be associated with survival [4, 5].

It has been suggested that a patient with 15% tumour shrinkage may have a better survival than a patient with 15% tumour growth, although both patients fall in the stable-disease category according to RECIST criteria. Therefore, the change in tumour size from baseline handled as a continuous variable, which differentiates such patients, might help to assess antitumor activity and to predict survival [6]. Data from phase I [7], II [8], and III [9, 10] clinical trials indicate that unidimensional continuous-scale tumour size (UCSTS) measurement is feasible and probably useful for assessing treatment effectiveness, particularly when the number of patients is small. However, UCSTS measurement may be difficult to perform. Further new lesions cannot be quantified numerically, and consequently are generally classified as PD or ignored.

Pharmacogenoscan is a large translational study aimed at associating the molecular profiles of tumour and blood samples with the response to first-line chemotherapy in patients with non-small-cell lung cancer (NSCLC). The primary objective of the study reported here was to compare the performance of UCSTS changes and RECIST 1.1 categories in predicting overall survival (OS) in the Pharmacogenoscan cohort.

## Methods

### Patients and study design

The Pharmacogenoscan study is a prospective study conducted in six hospitals in the Rhône-Alpes-Auvergne region of France to identify biological and histological factors associated with outcomes of patients with NSCLC. The study was approved by the ethics committee of the Grenoble University Hospital (NCT00222404), and all patients gave written informed consent before study inclusion.

Consecutive patientswith chemotherapy-naive NSCLC at any stage [11, 12] seen between July 2005 and August 2010 and having an ECOG-performance status (PS) [13] of 0 to 2 were included if they received platinum-based doublet chemotherapy as either neo-adjuvant or first-line treatment for metastatic or recurrent disease. All clinical data were recorded prospectively. Missing data were retrieved by one of us (ACT) before database lock.

**Figure 1:**
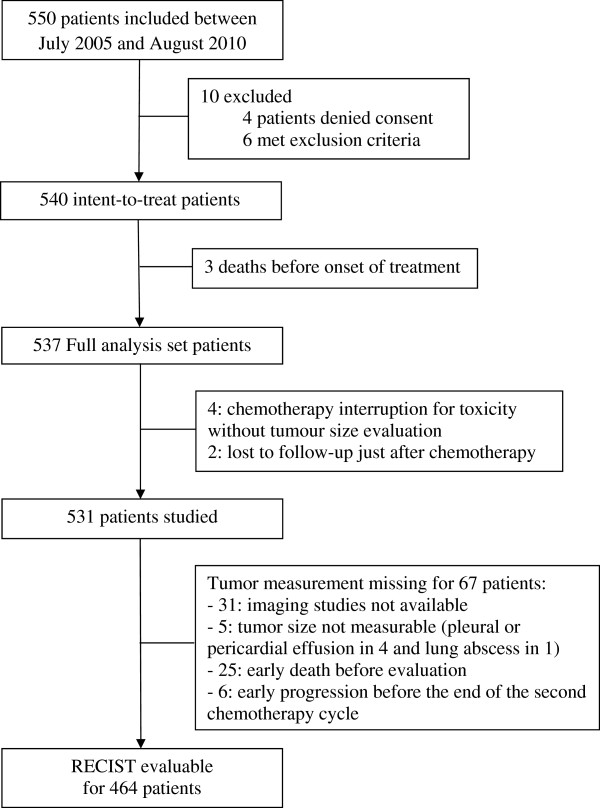
**Flow chart.**

As shown in the patient flow chart (Figure 1), we included 550 patients. We excluded 67 patients because of an inability to evaluate UCSTS changes or because of early death or disease progression. Landmark analysis [14, 15] was performed in these patients with a time point taken at 6 weeks. Finally, RECIST categories were available and studied for 464 patients.

### Tumour size evaluation

Baseline imaging was performed 20 days (25%-75% interquartile range (IQR), 12–31 days) before chemotherapy initiation, and the first follow-up evaluation occurred after two or three chemotherapy cycles (median, 42 days; IQR, 35–47 days) as decided by the investigator. Targets were measured on computed tomography (CT) images and reassessed for the purpose of the study by at least one physician specialised in thoracic oncology (ACT, DMS, PM, MP, PJS, PR, or PC). RECIST 1.1 response categories were determined [2]. For patients included before 2009, RECIST 1.0 categories were converted into RECIST 1.1 categories [16]. A systematic blind review of tumour response according to RECIST criteria was performed in a random sample of patients included by independent investigators belonging to different centres. The change in UCSTS over time was computed as follows: (UCSTS at first follow-up evaluation –UCSTS at baseline)/UCSTS at baseline. For the patients with at least one new lesion, we assigned a 100% increase in UCSTS measurement.

### Statistical analysis

Distributions of continuous variables were summarized by median (IQR) and of categorical variables by counts (percentages). Patients who were lost to follow-up by 1 December 2012 were considered censored. Follow-up duration was defined as the time from the first chemotherapy dose to last follow-up, and OS as the time from the first chemotherapy dose to death.

Kaplan-Meier curves of OS were plotted and compared between groups using the log-rank test. Univariate analyses were used to identify factors associated with OS. Variables associated with *P-*values lower than 0.20 by univariate analysis and those known to affect OSwere proposed to multivariate Cox models using a stepwise procedure. Variables associated with *p* < 0.05 in the multivariate context were kept in the models. The proportional hazard assumption was checked using martingale residuals. For this assumption to be plausible, we separately analysed early (<6 months) and late (≥6 months) deaths, using Cox models. Analyses were stratified for the ECOG-PS and the hospital. Hazard ratios (HRs) with their 95% confidence intervals (95% CIs) and *p* values were computed. We compared the non-nested RECIST and UCSTS models based on the Akaike Information Criterion (AIC).The AIC is maximum likelihood function penalised by the number of variables included in the model. It offers a relative estimate of the information lost when a given model is used to represent the process that generates the data. It defines the best model as the one with the lowest AIC value [17]. To evaluate the significance of the AICs’ difference, we calculated a chi-square (difference between -2log likelihood of both models) at a degree of freedom (difference between degrees of freedom of both models). Thus we obtained a *P*-value. All statistical analyses were performed using SAS 9.3 (SAS Institute, Cary, NC, USA).

## Results

### Patient characteristics

Figure 1 is the patient flow chart. Table 1 lists the main patient characteristics. The majority of patients had inoperable cancer (82%) and/or an ECOG-PS of 0 or 1 (96%). Various platinum-based chemotherapy doublets were used and 21 (5%) patients received a chemotherapy triplet including bevacizumab. Median follow-up duration was 13.7(IQR 7.2-28.1) months and median OS 14.2 (IQR, 7.3-28.9) months.

**Table 1:** **Univariate analysis (log rank test)**

Patient characteristics	Total (%) n = 464	Deceased (% of the total)	Median (IQR) survival (months)	***p***value
**Gender**				0.56
Male	353 (76)	292 (83)	14.2 (7.0-29.5)	
Female	111 (24)	100 (90)	14.2 (9.3-28.9)	
**Age (by quartile)**				0.84
< 54 years	112 (24)	96 (86)	13.7 (7.7-28.8)	
54-60 years	112 (24)	95 (85)	13.5 (8.4-30.1)	
60-67 years	117 (25)	96 (82)	15.6 (7.3-30.1)	
≥ 67 years	123 (27)	105 (85)	13.8 (6.9-27.9)	
**Performance status**				4.10^−4^
0	183 (39)	149 (81)	16.8 (10.3-35.5)	
1	261 (56)	224 (86)	12.2 (6.4-28.0)	
2	20 (4)	19 (95)	6.0 (2.8-13.9)	
**Charlson comorbidity index** [18]				0.71
0	378 (81)	321 (85)	13.6 (7.3-28.9)	
≥ 1	86 (19)	71 (83)	15.3 (7.3-29.5)	
**Histology**				<10^−4^
Adenocarcinoma	279 (60)	247 (89)	13.7 (7.1-27.9)	
Squamous cell carcinoma	112 (24)	79 (71)	20.2 (9.2-63.5)	
Large cell carcinoma	73 (16)	66 (90)	10.5 (6.3-20.2)	
**Cancer spread**				<10^−4^
Localised (II IIIA)	83 (18)	49 (59)	35.5 (18.5-not reached)	
Advanced (IIIB)	97 (21)	75 (87)	20.2 (10.8-41.5)	
Metastatic (IV)	284 (61)	268 (94)	10.5 (5.9-18.6)	
**Platinum-based doublet**				0.26
Platinum-gemcitabine	179 (39)	150 (84)	15.8 (8.0-33.2)	
Platinum-vinorelbine	77 (17)	64 (82)	15.2 (8.3-28.9)	
Platinum-docetaxel	74 (16)	64 (86)	12.7 (7.3-21.8)	
Platinum-paclitaxel	70 (15)	61 (87)	11.3 (4.3-26.9)	
Platinum-pemetrexed	64 (14)	54 (84)	16.0 (8.1-29.9)	
**Tumour response**				<10^−4^
Complete/partial response	146 (31)	108 (74)	20.8 (10.8-43.9)	
Stable disease	245 (53)	213 (87)	14.6 (8.2-28.8)	
Progressive disease	73 (16)	71 (97)	4.5 (2.7-8.3)	

IQR: interquartile range.

Median OS was 14.7 (IQR, 8.0-25.8) months in patients with no available CT scans and 15.8 (IQR, 10.2-31.6) months in those with non-measurable tumours.

### Tumour response

According to RECIST, 146 (31%) patients had a CR or PR, 245 (53%) SD, and 146 (31%) PD. The systematic review of the tumour response was performed for the first 64 (14%) patients and showed agreement with the initial evaluation in 60 (94%). The discrepancies were resolved by discussion.

**Figure 2:**
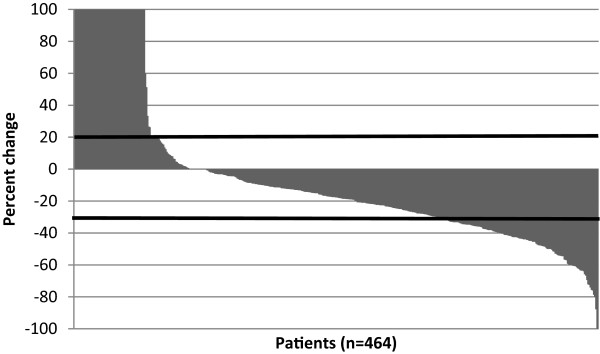
**Response according to percentage change in unidimensional continuous-scale tumour size (waterfall plot).**

Patients with measurable changes in tumour size had changes ranging from a 100% decrease to a 100% increase, with 347 (75%) showing at least some decrease according to RECIST (Figure 2).

**Figure 3:**
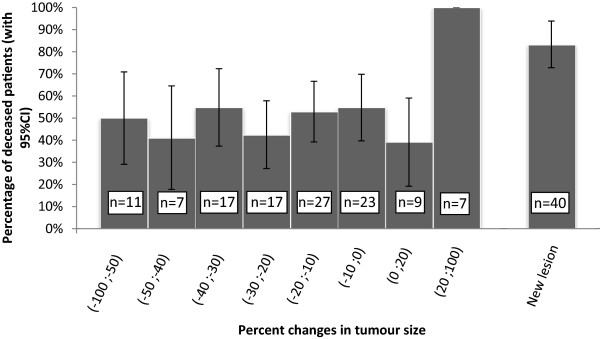
**1-year survival in patients with metastatic disease according to change in unidimensional continuous-scale tumour size.**

Figure 3 shows 1-year survival in patients with metastatic disease according to the change in UCSTS. In this group, 1-year mortality was about 50% for UCSTS changes between −100% and +20% (CR + PR + SD RECIST categories) and greater than 80% for UCSTS increases greater than 20% (PD RECIST category). In non metastatic tumours, predicted survival was related linearly with the logit of percentage of response without clear cut-off.

### Association between tumour response and overall survival (OS)

**Figure 4:**
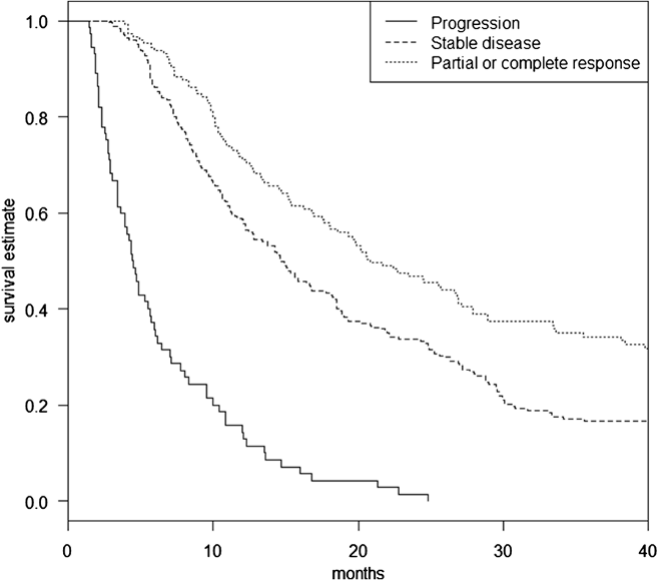
**Kaplan-Meier survival estimate according to RECIST.**

By univariate analysis (Table 1), ECOG-PS, histologic cancer type, and cancer spread (TNM classification) were significantly associated with OS. Response according to RECIST was associated with OS (*p* < 10^−4^, Figure 4).

Tables 2 and 3 show the results of the Cox models for the first 6 months and subsequent period, respectively. The analysis was routinely adjusted on histology and cancer spread. Sex and chemotherapy doublet were proposed to the model but not kept at the final step. The analysis was stratified on ECOG-PS as explained in the method, and on hospital. Accuracies as estimated by the AIC were better for RECIST 1.1 (*p* = 0.03) than for UCSTS, even after adjustment on confounders for early survival. However, no difference in accuracy was found between RECIST 1.1 and UCSTS for late survival (*p* = 0.15).

**Table 2:** **Akaike Information Criterion (AIC) values for the Cox models, First 6 months of follow-up (464 patients)**

Model		HR (95% CI)	***p***value	AIC*
RECIST	Partial response	1		575.762
	Stable disease	2.29 (1.06-4.94)	0.03	
	Progressive disease	18.48 (8.41-40.59)	<10^−4^	
UCSTS	per 10% difference	1.21 (1.16-1.26)	<10^−4^	578.585

The analysis was routinely adjusted on histology and cancer spread. Sex and chemotherapy doublet were proposed to the model but not kept at the final step. The analysis was stratified on ECOG-PS and hospital.

*In the model with RECIST, −2log likelihood = 563.762 with 6 degrees of freedom. In the model with UCSTS changes, −2log likelihood = 568.585 with 5 degrees of freedom.

*p* = 0.03.

HR, hazard ratio; 95% CI, 95% confidence interval; RECIST, Response Evaluation Criteria in Solid Tumors; UCSTS, unidimensional continuous-scale tumour size.

**Table 3:** **Akaike Information Criterion (AIC) values for the Cox models, Beyond the first 6 months of follow-up (370 patients)**

Model		HR (95% CI)	***p***value	AIC*
RECIST	Partial response	1		1835.264
	Stable disease	1.19 (0.90-1.58)	0.22	
	Progressive disease	3.12 (1.89-5.14)	<10^−4^	
UCSTS	per 10% difference	1.07 (1.03-1.11)	<10^−4^	1835.312

The analysis was routinely adjusted on histology and cancer spread. Sex and chemotherapy doublet were proposed to the model but not kept at the final step. The analysis was stratified on ECOG-PS and hospital.

*In the model with RECIST, −2log likelihood = 1823.264 with 6 degrees of freedom.In the model with UCSTS, −2log likelihood = 1825.312 with 5 degrees of freedom.

*p* = 0.15.

HR, hazard ratio; 95% CI, 95% confidence interval; RECIST, Response Evaluation Criteria in Solid Tumors; UCSTS, unidimensional continuous-scale tumour size.

## Discussion

In our study, tumour response to chemotherapy evaluated based on either RECIST or UCSTS was strongly associated with OS. UCSTS did not perform better than RECIST in predicting OS.We studied a large cohort of patients with NSCLC selected only based on ECOG-PS. Our population is representative of the NSCLC patients seen in daily practice. In particular, most patients had adenocarcinoma and 60% had metastatic disease.

RECIST 1.1 was superior over UCSTS in predicting early survival, probably due to the weight of poor prognosis among patients with PD. For predicting late survival, the two methods were similarly accurate. We are aware of a single previous study [19] of UCSTS versus WHO criteria for predicting survival. The patients had colorectal cancer and were given conventional chemotherapy. UCSTS did not perform better than WHO criteria with the three categories CR/PR, SD, and PD. One possible explanation is the variability in CT measurements of tumour size. In a study of 33 patients with NSCLC, the interobserver relative measurement change in unidimensional tumour size measurements varied from 0% to 194% [20]. In patients with a variety of thoracic and abdominal tumours, the results obtained by a single observer compared with multiple observers differed by more than 10% for 83% of lesions [21]. Finally, among patients with NSCLC, measurements on two CT scans obtained within less than 15 minutes often showed differences exceeding 1 or 2 mm [22].Thus, small changes in tumour size should be interpreted with caution. When using the WHO criteria,tumour measurement errors can be expected to produce objective response rates ranging from of 5% to 10% [23].Tumour response evaluation using RECIST appeared more reproducible. In our study, the review of results by a panel of experts found errorsin only 6% of patients. Andoh et al. [24] assessed the quality of radiology reports requiring RECIST and found that the combination of distributed educational materials and audit and feedback interventions improved radiology report quality by reducing the number of studies with errors from 30% to 22%.

Response rates (responders versus non-responders) do not include SD in the response category. Response is a common endpoint in phase III studies but performs poorly in predicting survival [25]. Patients can experience clinical benefits from treatment without a significant change in tumour size. Our results in the subgroup with metastatic disease suggest that a 20% increase in UCSTS (PD) may be a satisfactory cut-off for separating two prognostic groups. A study of patients with NSCLC [26] is consistent with this assumption: the 8-week rate of disease control, defined as no disease progression, performed better in predicting survival than did the 8-week rate of CR/PR. Recently, Mandrekar et al. [27] published the results from 13 trials including patients with metastatic cancer. They confirmed the utility of the RECIST-based response metrics. No alternative cut-offs or alternative categorical metrics appeared better than the RECIST standards.

One limitation of our study is the exclusion of 13% of the patients from the analysis. Most of these patients had early progression or death and were excluded to meet the conditions required for a landmark analysis [14, 15], i.e., to avoid bias in favour of responders. In our study we assigned a 100% increase in the UCSTS measurement for new lesions. They were excluded from the survival analysis, but finally, only 3% of patients had PD. We acknowledge that this way of expressing data may be over-simplistic. Furthermore, as the time of response evaluation was decided by each investigator (after 2 or 3 cycles of chemotherapy) the assessments were not performed within a 2-week window. Various chemotherapy doublets were used, and most patients (69%) received at least one subsequent line of chemotherapy, which may have attenuated the association between tumour shrinkage achieved with first-line chemotherapy and OS.

## Conclusion

In this large observational study of NSCLC patients given chemotherapy, UCSTS did not better perform than RECIST in predicting survival. In addition, our results suggest that distinguishing between PR and SD may be unhelpful for predicting survival of patients with metastatic disease. RECIST is easier to assess than UCSTS, which may deserve more specific assessment for trials with targeted therapies.

## Abbreviations

AIC: Akaike Information Criterion
CI: Confidence intervals
CR: Complete response
CT: COMPUTED tomography
HR: Hazard ratios
IQR: Interquartile range
NSCLC: Non-small-cell lung cancer
OS: Overall survival
PD: Progressive disease
PR: Partial response
PS: Performance status
RECIST: Response Evaluation Criteria in Solid Tumors
SD: Stable disease
UCSTS: Unidimensional continuous-scale tumour size.
